# Adiponectin Treatment Attenuates Cerebral Ischemia-Reperfusion Injury through HIF-1*α*-Mediated Antioxidation in Mice

**DOI:** 10.1155/2021/5531048

**Published:** 2021-07-14

**Authors:** Chan Zhang, Luming Zhen, Zongping Fang, Liang Yu, Yuanyuan Zhang, Haidong Wei, Junfeng Jia, Shiquan Wang

**Affiliations:** ^1^Outpatient Department, Xijing Hospital, The Fourth Military Medical University, Xi'an, Shaanxi 710032, China; ^2^Department of Anesthesiology, The Second Affiliated Hospital of Xi'an Jiaotong University, Xi'an, Shaanxi 710032, China; ^3^Department of Anesthesiology and Perioperative Medicine, Xijing Hospital, The Fourth Military Medical University, Xi'an, Shaanxi 710032, China; ^4^Department of Information, The Fourth Military Medical University, Xi'an, Shaanxi 710032, China; ^5^Department of Immunology, The Fourth Military Medical University, Xi'an, Shaanxi 710032, China

## Abstract

Adiponectin (ADPN) plays an important role in cerebral ischemia-reperfusion injury. Although previous studies have confirmed that ADPN pretreatment has a protective effect on ischemic stroke, the therapeutic effect of ADPN on ischemic stroke and the underlying mechanism are still unclear. In order to clarify these questions, focal transient cerebral ischemia was induced by middle cerebral artery occlusion (MCAO) in mice and ADPN was administered for three times at 6 h, 24 h, and 48 h after reperfusion. Meanwhile, a virus-delivered HIF-1*α* siRNA was used before ADPN administration. The infarct volume, neurological score, cellular apoptosis, and oxidative stress were assessed at 72 h after reperfusion. The long-term outcome of mice after stroke was recorded as well. The results indicated that ADPN treatment reduced the infarct volume (*P* = 0.032), neurological deficits (*P* = 0.047), cellular apoptosis (*P* = 0.041), and oxidative responses (*P* = 0.031) at 72 h after MCAO. Moreover, ADPN increased both the protein level and transcriptional activity of HIF-1*α* as evidenced by the transcription levels of VEGF (*P* = 0.046) and EPO (*P* = 0.043) at 72 h after MCAO. However, knockdown of HIF-1*α* partially reversed the antioxidant and treatment effect of ADPN after cerebral ischemia. In the observation of long-term outcome after ADPN treatment, it demonstrated that ADPN not only prevented the cerebral atrophy (*P* = 0.031) and the neurological function decline (*P* = 0.048), but also promoted angiogenesis (*P* = 0.028) after stroke. In conclusion, our findings suggest that ADPN is effective in treatment of ischemic stroke which could be attributed to the increased antioxidant capacity regulated by HIF-1*α*.

## 1. Introduction

Cerebrovascular disease, especially ischemic stroke, is still one of the leading causes of death and disabilities [1]. Although great progresses have been made in the treatment of ischemic stroke as thrombolysis by tissue-type plasminogen activator (t-PA) and endovascular treatment can rescue a few patients, more effective drugs and methods are still urgently needed for the rest of those unfortunate large populations who exceeded the very narrow therapeutic time window [2]. Therefore, more effective treatment methods have always been the goal of stroke study [3]. The adipocyte-secreted protein hormone adiponectin (ADPN) may have a potential therapeutic effect on stroke, as ADPN deficiency aggravates cerebral ischemia injury [4], while overexpression or supplementation of ADPN before stroke onset is protective [5, 6]. Human studies also indicate the important role of ADPN in stroke: the circulating ADPN was temporarily decreased in the acute phase of ischemic stroke in patients [7], and ADPN was negatively correlated with brain infarct volume and the severity of cerebral NIHSS score [8, 9]. Under physiological conditions, the amount of ADPN in the brain was very low, while in the process of cerebral ischemia injury, the circulating ADPN can enter the brain due to the destruction of the blood-brain barrier. ADPN plays multiple roles through ADPN receptors which are expressed in peripheral tissues and central nervous system. The main functions of ADPN include anti-inflammatory [10], antioxidant [11], and antiatherogenic properties [12]. A recent study confirmed the protective effect of exogenous ADPN pretreatment on ischemic stroke injury and found out that it was mediated through cAMP/PKA signaling [6]. However, the treatment effect of exogenous ADPN after stroke was undetermined, and the underlying mechanisms remain poorly understood.

The oxidative stress and inflammatory reaction are important pathological mechanisms of ischemic stroke. It is suggested that the antioxidant and anti-inflammatory strategy is one of the important methods for the treatment of ischemic stroke [13], such as 3-n butylphthalide, which is currently used in the treatment of stroke in China [14, 15]. A recent study interestingly revealed that ADPN also had antioxidant and anti-inflammatory functions when administrated before ischemic stroke, suggesting its clinical therapeutic potential for stroke [16]. However, the specific mechanism by which ADPN alleviates oxidative stress and inflammation was unclear. Hypoxia inducible factor-1 alpha (HIF-1*α*) is an important regulatory node in reducing oxidative stress and inflammation in stroke [17]. As we found its expression rapidly rises in the penumbra after ischemic stroke [18], we wonder whether HIF-1*α* was also involved in the treatment effect of ADPN after ischemic stroke.

Therefore, the present study is aimed at determining whether treatment with ADPN would attenuate cerebral ischemia-reperfusion injury through the increased antioxidant capacity dependent on HIF-1*α*.

## 2. Materials and Methods

### 2.1. Animals

Male C57 mice, 8-10 weeks old, weighing 20-25 g, were purchased from the Experimental Animal Center of Fourth Military Medical University (Xi'an, China). All the animals were housed in an environment with temperature of 22 ± 1°C, relative humidity of 50 ± 1%, and a light/dark cycle of 12/12 h. All animal studies (including the mice euthanasia procedure) were done in compliance with the regulations and guidelines of Fourth Military Medical University institutional animal care and according to the AAALAC and the IACUC guidelines.

### 2.2. Experimental Design

The study was divided into three main parts: the first part was to investigate the effectiveness of ADPN treatment on experimental stroke produced by middle cerebral artery occlusion (MCAO). Briefly, 80 mice were randomly allocated into four groups: sham group (mice subjected to sham operation), control group (mice subjected to MCAO), vehicle group (mice subjected to MCAO and treated with vehicle at 6 h, 24 h, and 48 h after reperfusion), and ADPN group (mice subjected to MCAO and treated with ADPN at 6 h, 24 h, and 48 h after reperfusion). The dose of ADPN was 5 mg/kg via tail vein injection according to a previous study [6]. Each group was composed of 20 mice: 8 mice were used for evaluation of neurological score and infarct volume, 4 for terminal deoxynucleotidyl transferase-mediated 2′-deoxyuridine 5′-triphosphate nick-end labeling (TUNEL) and immunofluorescence staining, and 4 for biochemical tests and 4 for western blotting analysis. The neurological score, infarct volume, TUNEL-positive neurons, cleaved caspase-3, HIF-1*α*, erythropoietin (EPO), and vascular endothelial growth factor (VEGF) protein levels were analyzed at 72 h after reperfusion. In addition, the cellular localization of HIF-1*α* was examined by immunofluorescence staining.

The second part of the experiment was to explore the role of HIF-1*α* in ADPN treatment after cerebral ischemia-reperfusion by using a virus-delivered HIF-1*α* siRNA. In this part, 60 mice were randomized into three groups: ADPN, control siRNA, and HIF-1*α* siRNA groups. ADPN group was the same as the first part. For control siRNA group, mice were treated with stereotactic injection of virus recombined with control siRNA 3 weeks before MCAO, and then, ADPN was injected via tail vein at 6 h, 24 h, and 48 h after reperfusion, and for HIF-1*α* siRNA group, mice were treated with stereotactic injection of virus recombined with HIF-1*α* siRNA 3 weeks before MCAO, and then, ADPN was injected via tail vein at 6 h, 24 h, and 48 h after reperfusion. Each group was composed of 20 mice: 8 were used for neurological scoring and infarct volume evaluation, 4 for biochemical tests and 4 for western blotting, and 4 for TUNEL staining. The neurological score, brain infarct volume, cellular apoptosis by TUNEL staining and cleaved caspase-3, and oxidation products were assessed at 72 h after reperfusion.

The third part was to assess the long-term outcome and angiogenesis of mice treated with ADPN after cerebral ischemia. All 32 mice were randomized into two groups: ADPN and vehicle groups. The treatment was the same as the first part. Each group had 16 mice: 8 were used for neurological scoring and brain atrophy evaluation, 4 for immunofluorescence staining, and 4 for western blotting analysis of von Willebrand factor (vWF) 28 days after MCAO.

### 2.3. Focal Cerebral Ischemia and Reperfusion

Mice were allowed for free access to food and tap water before surgery. Cerebral ischemia was induced by MCAO as previously described [19]. Briefly, the mice were anesthetized with 1.5% isoflurane. A silicon-coated suture (RWD Life Science) was then inserted into the right external carotid artery and advanced through the internal carotid artery to obstruct the MCA. The suture remained in position for 1 h during the arterial occlusion and was then removed to allow subsequent reperfusion. The body temperature of the mice was monitored by a rectal probe and maintained at 37 ± 0.5°C by using a heating pad. A laser Doppler sensor for blood flow monitoring was placed on the surface of the skull (2 mm caudal and 4 mm lateral to the bregma). A procedure with 80% decrease and 70% recovery of the regional cerebral blood flow was considered to be a successful ischemic injury.

### 2.4. Assessment of the Neurological Deficit

Based on the scoring system of Garcia et al. [20], the neurological behavior of the mice was assessed 72 h and 28 d postreperfusion by an observer blind to the animal grouping. This system consisted of the following six tests: spontaneous activity, symmetrical movements of the upper and lower limbs, forepaw outstretching, climbing, body proprioception, and response to vibrissal touch. The final score was the sum of all six individual test scores. The minimum neurological score was 3, while the maximum was 18.

The grid-walking test was used to assess the walking performance of the mice. The grid apparatus (40 × 20 cm^2^, each grid cell 2 × 2 cm^2^, and height 50 cm) was located in a sound attenuated room. Performance was recorded for 60 s using a video camera located beside the apparatus at an angle of approximately 20 to 40 degrees. A foot slip was recorded when one paw completely missed a bar with the limb falling between the bars or when the paw was correctly placed on the bar but slipped off during weight bearing. The total steps of the left forelimb and hind limb were counted, and the percentage of foot fault was measured by dividing the number of foot slips of the left forelimb and left hind limb by the total number of left steps taken with 60 s [21].

### 2.5. Measurement of Infarct Size

After mice were euthanized, the brains were removed and the ones with clot formation and/or subarachnoid hemorrhage were eliminated. The brains were first sectioned into 1 mm slices, incubated in a 2% solution of 2,3,5-triphenyltetrazolium chloride (MP Biomedicals) at 37°C for 15 min, and then fixed in 4% formalin. The stained sections were photographed using a digital camera and measured in a blinded manner with an image analysis software. The total volumes of both contralateral (vc) and ipsilateral hemisphere (vl) were measured, and the infarct percentage (%I) was calculated using the following formula: %I = 100∗(vc − vl)/vc, to avoid mismeasurement secondary to edema.

### 2.6. Brain Atrophy Measurement

Mice were anesthetized and then perfused with ice-cold saline, followed by fixation with 4% paraformaldehyde. The brains were then cut into 12 *μ*m thick coronal sections. Nissl staining was carried out according to manufacturer instructions. Briefly, the sections were washed with PBS for 3 times. All sections were stained with 1% methylviolet for 10 min. The sections were rinsed with double distilled water several times. Graded ethanol and xylenes were then used to treat the sections. Finally, the sections were imaged under a light microscope. The atrophy volumes were counted by investigators who were blinded to the grouping in our study. The formula was contralateral hemisphere brain area minus infarct hemisphere area to contralateral hemisphere brain area.

### 2.7. Microvessel Counts

Six 12 *μ*m thick coronal sections were stained with vWF from 0.5 mm anterior to 0.5 mm posterior in bregma. The number of microvessels was carried out using a 10x objective lens. The microvessels with a well-defined linear vessel shape were counted. The number of microvessels was obtained from the averaged number of six sections.

### 2.8. Transfection of HIF-1*α* siRNA

The recombinant adeno-associated virus 9 (AAV) containing HIF-1*α* siRNA-mCherry or control siRNA-mCherry was purchased from Genechem Co., Ltd. (Shanghai, China). The target sequence was 5′-CACCAU GAU UUU ACU ATT-3′, and the control sequence was 5′-UUC UCC GAA CGU GUC ACG-3′. Transfection was processed by stereotactic injection. The injection coordinates were 0.4 mm anterior to bregma, 1.0 mm lateral to the midsagittal line, and 1.5 mm deep from the cranial surface. Three weeks after injection, the reliability of siRNA was determined by immunofluorescence labeling and western blotting, as shown in Supplementary Figure S1.

### 2.9. Terminal Deoxynucleotidyl Transferase dUTP Nick-End Labeling (TUNEL) Staining

Cellular apoptosis was evaluated at 72 h after reperfusion. TUNEL staining was performed using an in situ cell death detection kit (Roche Diagnostics, Mannheim, Germany) according to the manufacturer's instructions. Mice brains were fixed with 4% paraformaldehyde. The tissue was then cut into 12 *μ*m thick coronal sections from 0.5 mm prior to bregma. Three slices from each mouse were used for TUNEL staining. Three fields from the penumbra zone for each slice were observed using a 40x objective lens. The ratio of TUNEL-positive cells to the number of total cells was considered as the apoptosis index and obtained by an observer blinded to the grouping. The ischemic penumbra area was defined as previously described [19].

### 2.10. Immunofluorescence Staining

Mice were perfused and fixed with 4% paraformaldehyde at 72 h after reperfusion. After dehydration with 30% sucrose, the brain was frozen and then cut into 12 *μ*m sections (approximately 1.33 mm from the rostral to the bregma). The slices were then washed with PBS, incubated with 0.3% Triton X-100 for 5 min at room temperature, and consequently blocked with 5% fetal bovine serum (BSA) for additional 30 min. Slices were incubated with rabbit anti-HIF-1*α* antibody (1 : 200, Abcam, Cambridge, London, UK) and rabbit anti-vWF antibody (1 : 100, Abcam, Cambridge, London, UK) at 4°C overnight, consequently probed with Alexa Fluor 488-conjugated donkey anti-rabbit antibody (1 : 400, Abcam, Cambridge, London, UK) and NeuroTrace red (1 : 2000, Molecular Probes; a dye for labeling of neurons based on Nissl stain), and finally visualized under a microscope (OLYMPUS, BX51) using the DP2-BSW software. Three fields from the penumbra zone for each slice were observed using a 40x objective lens.

### 2.11. Western Blot Analysis

The brain tissue of the ischemic penumbra was dissected at 72 h after reperfusion. All samples were homogenized in a RIPA lysis buffer (Beyotime, Nantong, China) containing whole proteinase inhibitor cocktail on ice. A BCA protein assay kit (Beyotime, Nantong, China) was used to determine the protein concentration. An equivalent amount of protein (30 *μ*g per lane) was loaded and separated by a 12% SDS-PAGE gel. After electrophoresis, the protein was transferred to a polyvinylidene difluoride membrane. The membrane was blocked using 2% BSA in TBST. After overnight incubation at 4°C with the primary antibodies HIF-1*α* (1 : 1000, Abcam, Cambridge, London, UK), EPO (1 : 1000, Abcam, Cambridge, London, UK), VEGF (1 : 1000, Abcam, Cambridge, London, UK), vWF (1 : 1000, Abcam, Cambridge, London, UK), and *β*-actin (1 : 1000. Abcam, Cambridge, London, UK), the membrane was incubated with horseradish peroxidase-conjugated secondary goat anti-rabbit antibody (1 : 5000, Pierce, Rockford, IL) for 1 h at room temperature. Optical density of each band was quantified using the ImageJ software. The dissection of ischemic penumbra was as previously described [22]. Briefly, the brain was coronally cut into three slices: the first cutting was 2 mm from the anterior tip of the frontal lobe, and the second cutting was 4 mm from the first cutting. The middle part that corresponded to the ischemic core and penumbra was dissected. The midline between the two hemispheres was identified, and a longitudinal cut (from top to bottom) approximately 1 mm from the midline through infarct hemisphere was made. We then made a transverse diagonal cut at approximately the 2-o'clock position to separate the core (i.e., striatum and overlying cortex) from the penumbra (adjacent cortex).

### 2.12. Biochemical Estimations

At 72 h after reperfusion, the penumbra was dissected and homogenized in cold sodium chloride after it has been weighted. The homogenate was then centrifuged at 10000 × g for 15 min, and the supernatant was collected and consequently frozen at -80°C for further analysis. The protein content was measured using BCA protein assay kit (Beyotime, Nantong, China).

Assay kits were used to measure the content of oxidation products (MDA, 8-OHdG, protein carbonyl, and protein nitrotyrosine). The carbonyl kit and the nitrotyrosine kit were purchased from CHEMICON. Others were purchased from Nanjing Jiancheng Bioengineering Institute. The assays were done according the manufacturer's instructions.

### 2.13. Quantitative Reverse Transcription Polymerase Chain Reaction (qRT-PCR) Measurement

Total RNA was extracted from the penumbra of the cerebral cortex with Trizol reagent (Life Technologies, USA) according to the manufacturer's instructions. The cDNA was subsequently synthesized with a standard cDNA synthesis kit (Life Technologies, USA). The mRNA level of *β*-actin was used as an internal control. The sequences of primers were as follows: VEGF, forward: 5′-GCACTGGACCCTGGCTTTACT-3′ and reverse: 5′-ACTTCACCACTTCATGGGTCTTGTG-3′; HIF-1*α*, forward: 5′-GTTACAGGATTCCAGCAGACC-3′ and reverse: 5′-TGGGTAGAAGGTGGAGATGC-3′; EPO, forward: 5′-AATGGAGGTGGAAGAACAGG-3′ and reverse: 5′-ACCCGAAGCAGTGAAGTGA-3′.

The reverse transcription reaction was carried out in a 20 *μ*l volume with 500 ng of total RNA, 16°C for 30 min, 42°C for 42 min, and 85°C for 5 min. The PCR cycling began with template denaturing at 95°C for 5 min, followed by 40 cycles of 95°C for 10 s, 60°C for 20 s, 72°C for 20 s, and 78°C for 20 s.

### 2.14. Statistical Analysis

Data were presented as mean ± standard error. Statistical analysis was performed using the Statistical Package for the Social Sciences (SPSS) version 16.0 for Windows, except for neurological scores which were expressed as median with interquartile range and consequently analyzed by Kruskal-Wallis tests. The Bonferroni test was used as a correction method. Other values were analyzed by one-way ANOVA, followed by post hoc Tukey test. A *P* value less than 0.05 was considered statistically significant.

## 3. Results

### 3.1. ADPN Treatment Reduced Cerebral Ischemia-Reperfusion Injury

**Figure 1:**
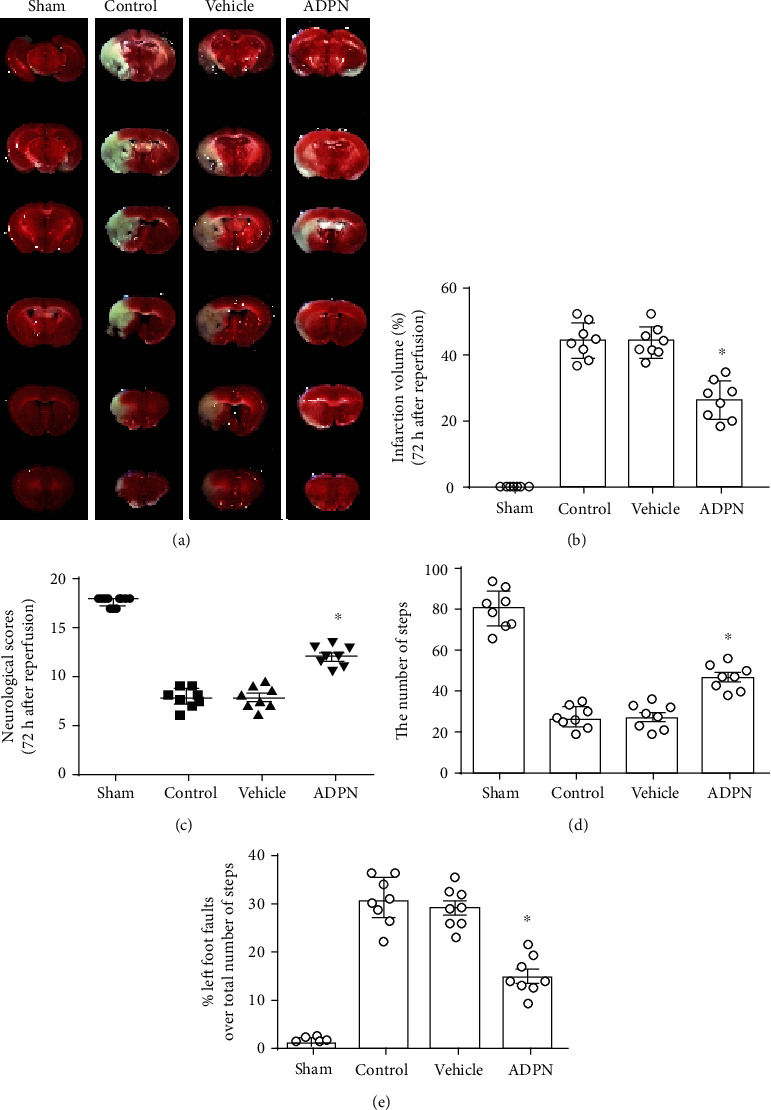
ADPN treatment exerted neuroprotective effect against cerebral ischemia injury in mice. (a) Representative photographs of brain slices showing infract volume assessed 72 h after reperfusion in mice. (b) The infarct volumes as percentages of the contralateral hemisphere are presented as the mean ± SD and analyzed by one-way ANOVA with Tukey's posttest. (c) The scoring system of Garcia et al. was evaluated 72 h after reperfusion. The data are presented as the median with range and analyzed by the Kruskal-Wallis test followed by Dunn's test. (d) The total steps were assessed at 72 h after reperfusion. (e) The error ratio was assessed at 72 h after reperfusion. ^∗^*P* < 0.05 compared to the vehicle group. *n* = 8 per group.

The effect of ADPN administration on infarct volume and neurological deficit in mice were observed at 72 h after cerebral ischemia. The infarct volumes were notably decreased in ADPN group when compared to vehicle group (44.3% ± 5.1% vs. 26.2% ± 4.5%, *P* < 0.05, Figures 1(a) and 1(b)). The neurological score was increased in the ADPN group when compared to vehicle group [12 (2) vs. 8.5 (1.5), *P* < 0.05, Figure 1(c)]. The grid-walking test showed that ADPN increased the total steps (21 ± 5.2 vs. 58 ± 6.3, *P* < 0.05, Figure 1(d)) and reduced the error ratio (12.7% ± 3.2% vs. 28.7% ± 5.6%, *P* < 0.05, Figure 1(e)). There is no statistical difference between the control group and vehicle group in infarct volume, neurological score, and step error.

### 3.2. ADPN Treatment Inhibited Apoptosis in Cerebral Ischemic Penumbra

**Figure 2:**
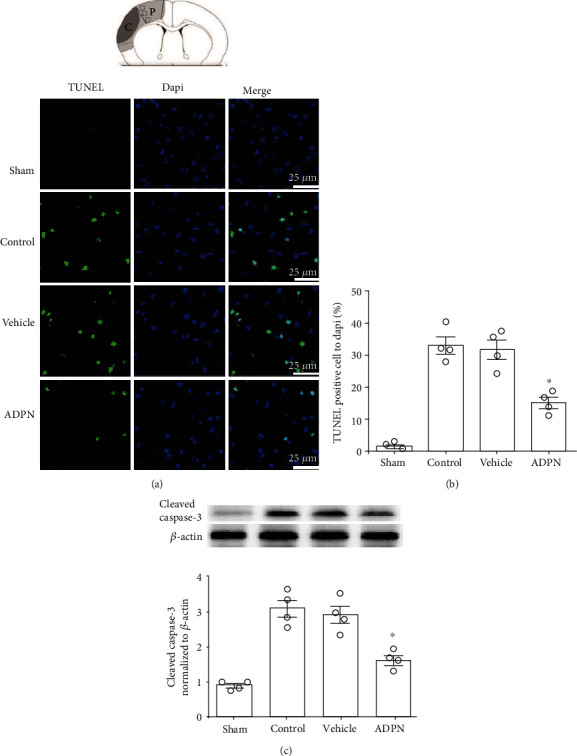
ADPN treatment alleviated apoptosis in the ischemic penumbra. (a) Representative photomicrographs showing TUNEL staining in the ischemic penumbra of mice at 72 h after reperfusion. (b) The percentages of TUNEL-positive cells in the ischemic penumbra. (c) Cleaved caspase-3 level in the ischemia penumbra. Data are presented as the mean ± SD and analyzed by the *Kruskal-Wallis* test followed by *Dunn's* test. ^∗^*P* < 0.05 compared to vehicle group, *n* = 4.

Cellular apoptosis was analyzed to confirm the neuroprotective effect of ADPN. Representative photomicrographs of TUNEL staining in the ischemic penumbra area are shown in Figure 2(a). Compared to vehicle groups, ADPN induced a reduction in TUNEL-positive cells (18.9 ± 4.9% vs. 35.4 ± 4.6%, *P* < 0.05), while no significant difference in the number of TUNEL-positive neurons was observed between the control and vehicle groups at 72 h postreperfusion (*P* > 0.05), as shown in Figure 2(b). Furthermore, levels of cleaved caspase-3 were analyzed by western blotting at 72 h after reperfusion. Compared to vehicle group, ADPN treatment decreased the levels of cleaved caspase-3 (1.7 ± 0.3 vs. 3.1 ± 0.4, *P* < 0.05); in addition, no difference was observed between the control group (2.9 ± 0.4 vs. 3.1 ± 0.4, *P* > 0.05) and vehicle group, as shown in Figure 2(c).

### 3.3. ADPN Treatment Mitigated Oxidative Stress after Reperfusion

**Figure 3:**
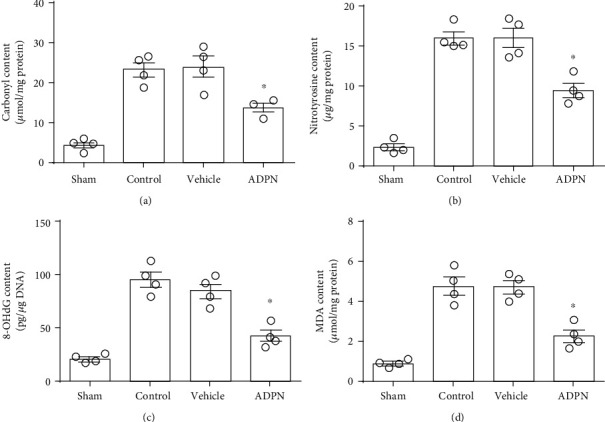
The effect of ADPN on oxidative products. (a) The content of carbonyl protein in the ischemic penumbra. (b) The content of nitrotyrosine protein in the ischemic penumbra. (c) The content of 8-OHdG in the ischemic penumbra. (d) The content of MDA in the ischemic penumbra. Data are presented as the mean ± SD and analyzed by the *Kruskal-Wallis* test followed by *Dunn's* test. ^∗^*P* < 0.05 compared to vehicle group, *n* = 4 per group.

To further determine whether exogenous ADPN could reduce the oxidative stress injury, indicators of protein oxidation (carbonyl protein and nitrotyrosine protein contents) and indicators of DNA injury 8-OHdG and lipid peroxidation MDA were analyzed, respectively, in ischemic penumbra at 72 h postreperfusion (Figure 3). Compared with the vehicle group, ADPN treatment reduced all these factors: the carbonyl protein content (14.8 ± 2.8 *μ*mol/mg vs. 23.0 ± 3.1 *μ*mol/mg, *P* < 0.05, Figure 3(a)), the nitrotyrosine protein content (9.4 ± 1.3 *μ*g/mg vs. 15.9 ± 1.9 *μ*g/mg, *P* < 0.05, Figure 3(b)), the 8-OHdG content (41.7 ± 9.1 pg/mg DNA vs. 84.2 ± 11.7 pg/mg DNA, *P* < 0.05, Figure 3(c)), and the MDA content (2.2 ± 0.5 *μ*g/mg protein vs. 4.7 ± 0.5 *μ*g/mg protein, *P* < 0.05, Figure 3(d)). There was no significant difference between the vehicle group and control group: the carbonyl protein content (23.0 ± 3.1 *μ*mol/mg vs. 23.7 ± 4.5 *μ*mol/mg, *P* > 0.05, Figure 3(a)), the nitrotyrosine protein content (15.9 ± 1.9 *μ*g/mg vs. 15.3 ± 1.2 *μ*g/mg, *P* > 0.05, Figure 3(b)), the 8-OHdG content (84.2 ± 11.7 pg/mg DNA vs. 94.9 ± 14.3 pg/mg DNA, *P* > 0.05, Figure 3(c)), and the MDA content (4.7 ± 0.5 *μ*g/mg protein vs. 4.9 ± 0.7 *μ*g/mg protein, *P* > 0.05, Figure 3(d)).

### 3.4. ADPN Increased the Neuronal Expression of HIF-1*α* after Reperfusion

**Figure 4:**
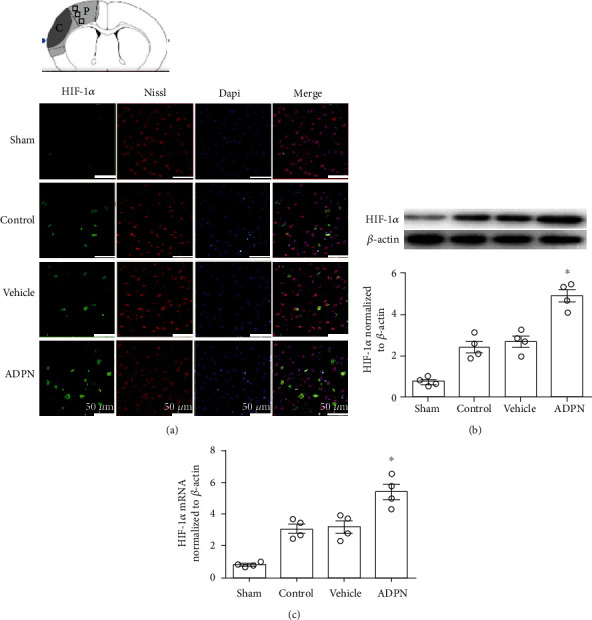
ADPN enhanced the proteins and mRNA expression of HIF-1*α* at 72 h after reperfusion. (a) Representative photomicrographs of HIF-1*α* staining in the ischemic penumbra. (b) Western blotting analysis of HIF-1*α* protein expression. (c) The RT-PCR analysis of HIF-1*α* mRNA. Data are presented as the mean ± SD and analyzed by the *Kruskal-Wallis* test followed by *Dunn's* test. ^∗^*P* < 0.05 compared to vehicle group, *n* = 4 per group.

Spatial localization of HIF-1*α* was determined using immunofluorescence staining. Furthermore, the protein level of HIF-1*α* was measured by western blotting, and the mRNA level of HIF-1*α* was tested by qRT-PCR (Figure 4). As shown in Figure 4(a), HIF-1*α* was mainly expressed in neurons among different groups. Higher HIF-1*α* protein level was observed after administration of ADPN compared to vehicle group (5.4 ± 0.8 vs. 3.2 ± 0.7, *P* < 0.05), while no significant difference was found between the vehicle group and control group (3.2 ± 0.7 vs. 3.0 ± 0.5, *P* > 0.1) at 72 h postreperfusion (Figure 4(b)). Moreover, compared to vehicle group (3.8 ± 0.5), exogenous ADPN induced an increase of HIF-1*α* mRNA (5.4 ± 0.7, *P* < 0.05), while there was no difference between the control group and vehicle group (*P* > 0.05).

### 3.5. ADPN Increased the Expression of EPO and VEGF after Reperfusion

**Figure 5:**
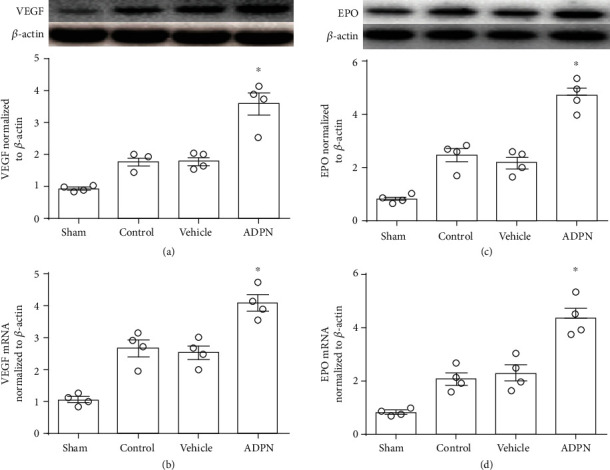
ADPN enhanced the proteins and mRNA expression of VEGF and EPO at 72 h after reperfusion. (a) Western blotting analysis of EPO protein expression. (b) The RT-PCR analysis of EPO mRNA. (c) Western blotting analysis of VEGF protein expression. (d) The RT-PCR analysis of VEGF mRNA. Data are presented as the mean ± SD and analyzed by the *Kruskal-Wallis* test followed by *Dunn's* test. ^∗^*P* < 0.05 compared to vehicle group, *n* = 4 per group.

The expression of HIF-1*α* target genes EPO and VEGF, which represented the transcriptional activity of the HIF-1*α*, was analyzed as shown in Figure 5. Compared to vehicle group, ADPN not only increased VEGF protein level (4.6 ± 0.5 vs. 2.2 ± 0.4, *P* < 0.05, Figure 5(a)) and EPO protein level (5.1 ± 0.7 vs. 2.6 ± 0.4, *P* < 0.05, Figure 5(c)), but also increased the mRNA level of VEGF (4.4 ± 0.6 vs. 2.3 ± 0.5, *P* < 0.05, Figure 5(b)) and EPO (4.1 ± 0.4 vs. 2.5 ± 0.4, *P* < 0.1, Figure 5(d)). Both the protein level and mRNA level of EPO and VEGF had no difference between the control group and vehicle group.

### 3.6. Knockdown of HIF-1*α* Partly Reversed the Protective Effect of ADPN

**Figure 6:**
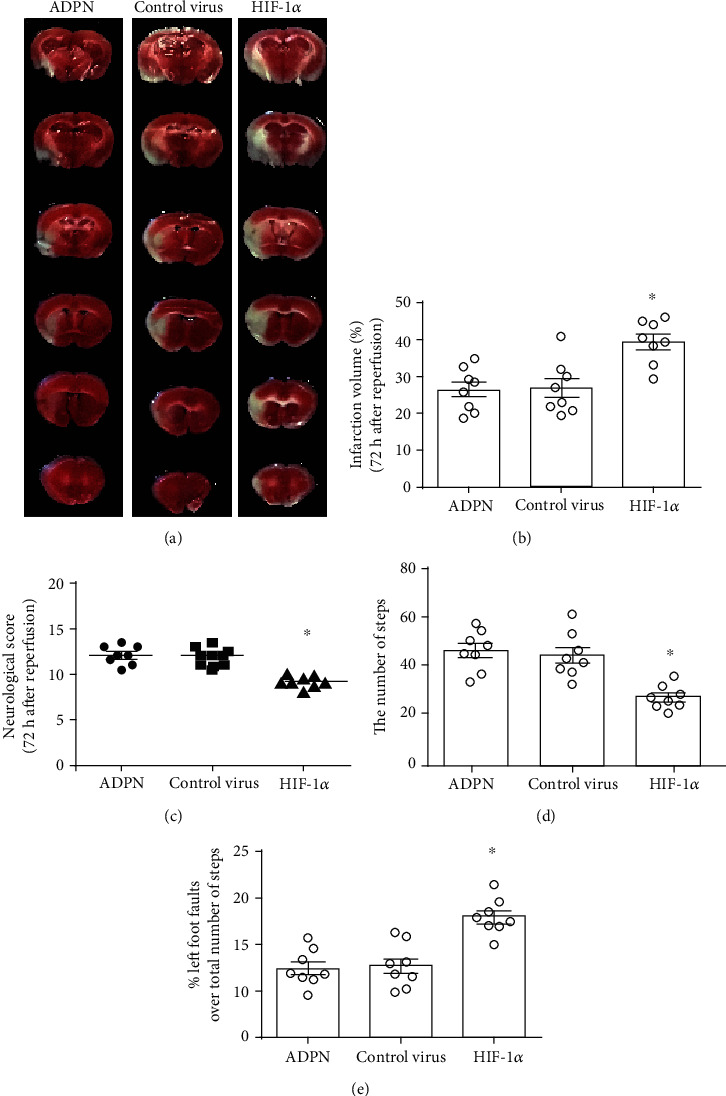
Downregulating HIF-1*α* with AAV-delivered siRNA can reduce the protective effect of ADPN. (a) Representative photographs of brain slices showing infract volume assessed 72 h after reperfusion in mice. (b) The infarct volumes as percentages of the contralateral hemisphere are presented as the mean ± SD and analyzed by one-way ANOVA with Tukey's posttest. (c) The scoring system of Garcia et al. was evaluated 72 h after reperfusion. The data are presented as the median with range and analyzed by the *Kruskal-Wallis* test followed by *Dunn's* test. (d) The total steps were assessed at 72 h after reperfusion. (e) The error ratio was assessed at 72 h after reperfusion. ^∗^*P* < 0.05 compared to the ADPN group. *n* = 8 per group.

**Figure 7:**
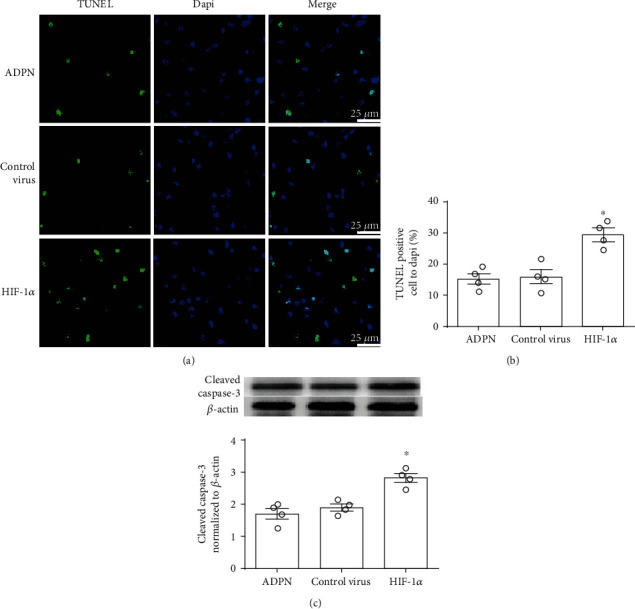
Downregulating HIF-1*α* with AAV-delivered siRNA reversed the antiapoptotic effect of ADPN. (a) Representative photomicrographs showing TUNEL staining in the ischemic penumbra of mice at 72 h after reperfusion. (b) The percentages of TUNEL-positive cells in the ischemic penumbra. (c) Cleaved caspase-3 level in the ischemia penumbra. Data are presented as the mean ± SD and analyzed by the *Kruskal-Wallis* test followed by *Dunn's* test. ^∗^*P* < 0.05 compared to ADPN group, *n* = 4.

**Figure 8:**
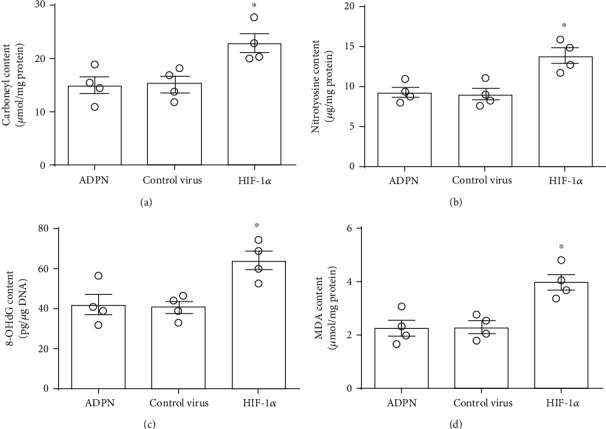
Downregulating HIF-1*α* with AAV-delivered siRNA reversed the antioxidant effect of ADPN. (a) The content of carbonyl protein in the ischemic penumbra. (b) The content of nitrotyrosine protein in the ischemic penumbra. (c) The content of 8-OHdG in the ischemic penumbra. (d) The content of MDA in the ischemic penumbra. Data are presented as the mean ± SD and analyzed by the *Kruskal-Wallis* test followed by *Dunn's* test. ^∗^*P* < 0.05 compared to ADPN group, *n* = 4 per group.

In order to investigate the role of HIF-1*α* in exogenous ADPN-induced cerebral protection after experimental stroke, the recombinant AAV containing HIF-1*α* siRNA was applied. The neurological score, the infarct volume, TUNEL-positive cells, and the indicators of oxidative stress were measured. Compared to control virus, neurological score was lower in HIF-1*α* siRNA group [9 (1) vs. 12 (1.5), *P* < 0.1, Figure 6(c)]; the infarct volume was larger (39.0% ± 5.5% vs. 27.2% ± 6.7%, *P* < 0.05, Figure 6(b)); moreover, the total steps were decreased (52 ± 6.5 vs. 29.1% ± 4.3, *P* < 0.05, Figure 6(d)), and the error ratio was increased (23.0% ± 6.5% vs. 13.1% ± 7.3%, *P* < 0.05, Figure 6(e)). Meanwhile, HIF-1*α* siRNA group had more apoptotic cells (19.6% ± 6.5% vs. 28.7% ± 5.2%, *P* < 0.05, Figure 7(b)) and higher levels of cleaved caspase-3 (2.8 ± 0.5 vs. 1.9 ± 0.3, *P* < 0.1, Figure 7(c)) and worsened oxidative stress injury: the carbonyl protein content (20.1 ± 1.8 *μ*mol/mg vs. 15.0 ± 2.4 *μ*mol/mg, *P* < 0.05, Figure 8(a)), the nitrotyrosine protein content (13.2 ± 1.3 *μ*g/mg vs. 8.9 ± 1.3 *μ*g/mg, *P* < 0.05, Figure 8(b)), the 8-OHdG content (63.8 ± 8.4 pg/mg DNA vs. 40.2 ± 5.1 pg/mg DNA, *P* < 0.05, Figure 8(c)), and the MDA content (4.0 ± 0.5 *μ*g/mg protein vs. 2.3 ± 0.4 *μ*g/mg protein, *P* < 0.05, Figure 8(d)).

### 3.7. ADPN Improved Long-Term Neurological Behavioral and Promoted Angiogenesis

**Figure 9:**
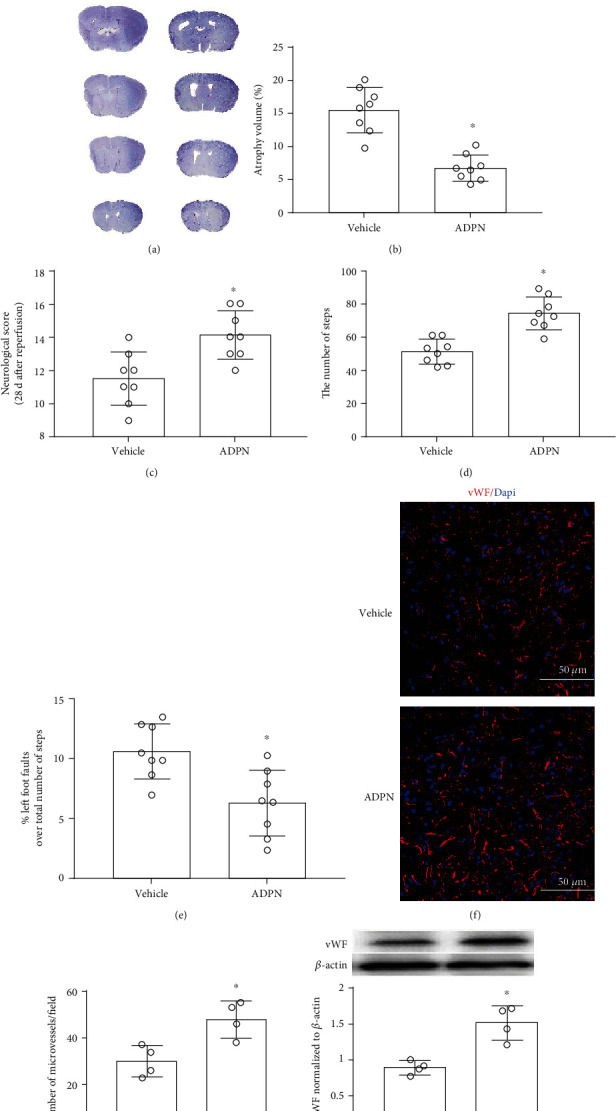
ADPN promoted angiogenesis and improved long-term neurobehavioral outcome. (a) Representative photomicrographs of atrophy volume. (b) The atrophy volume as percentages of the contralateral hemisphere is presented as the mean ± SD and analyzed by one-way ANOVA with Tukey's posttest. (c) The scoring system of Garcia et al. was evaluated on day 28 after reperfusion. The data are presented as the median with interquartile range and analyzed by the *Kruskal-Wallis* test followed by *Dunn's* test. (d) The total steps on day 28 after reperfusion. (e) The error ratio on day 28 after reperfusion. (f) Representative photomicrographs of angiogenesis. (g) Microvessel density statistics. (h) Western blotting analysis of vWF protein expression. These data except for the Garcia et al. score are presented as the mean ± SD and analyzed by one-way ANOVA with Tukey's posttest. ^∗^*P* < 0.05 compared to the vehicle group. *n* = 8 per group.

To investigate the effect of ADPN on long-term brain injury after MCAO, the brain atrophy volume and the behavioral deficits was assessed 28 days after MCAO. Quantitative analysis revealed that brain atrophy was prevented in ADPN group compared with vehicle group (6.4 ± 1.1% vs. 15.1 ± 1.6%, *P* < 0.05, Figure 9(b)), and similar results were seen in scoring system of Garcia et al. [14 (2) vs. 11.5 (1.5), *P* < 0.05, Figure 1(c)]. Meanwhile, compared to the vehicle group, the ADPN group demonstrated significant improvement in performance of grid-walking test: the total steps were increased (72 ± 12 vs. 53 ± 9, *P* < 0.05, Figure 9(d)), and the error ratio was decreased (10.7% ± 2.2% vs. 5.8% ± 1.6%, *P* < 0.05, Figure 9(e)). To examine the effect of ADPN on angiogenesis after MCAO, the number of microvessels was counted. The number of vWF-stained microvessels in the ADPN group mice on the 28 day after MCAO was more than that in vehicle group (30 ± 5.6 vs. 50 ± 6.1, *P* < 0.05, Figure 9(g)). Similarly, the level of vWF protein in ADPN group was increased than that in vehicle group (1.4 ± 0.3 vs. 0.9 ± 0.3, *P* < 0.05, Figure 9(h)).

## 4. Discussion

The present study showed that ADPN treatment is effective in reducing cerebral ischemia-reperfusion injury in a mouse stroke model. Briefly, the administration of ADPN improved neurological scores, reduced infarct volume, ameliorated neuronal apoptosis, and decreased oxidative products. Such therapeutic effect of ADPN on stroke could be attributed to the boosting of antioxidant capacity by HIF-1*α* in neurons. Moreover, we also found that ADPN could improve the long-term neurobehavioral outcome and promote angiogenesis in the ischemic penumbra after stroke.

ADPN is mainly produced and secreted by adipose tissue. It can also be synthesized and secreted by nonadipose tissues such as murine cardiomyocytes and human liver. ADPN is known to have antioxidant, antidiabetic, and anti-inflammatory properties, which can confer protection for chronic diseases [11]. Recent studies have identified the association between hypoadiponectinemia and increased mortality following ischemia stroke, as well as the negative correlation between ADPN levels and primary infarct size [23], suggesting ADPN has an important role in stroke. However, under physiological conditions, the amount of APDN in the brain is very low [24]. Nevertheless, the content of ADPN increases in ischemic hemisphere after stroke, while no ADPN mRNA was found in the ischemic hemisphere and contralateral hemisphere [25]. This indicates that the circulating ADPN could enter the brain tissue through injured BBB after ischemia [26], highlighting the feasibility of exogenous ADPN in stroke treatment. Indeed, exogenous ADPN injected before MCAO had protective effect on infarct size and neurological deficit scores [5]. This was consistent with the results obtained by Bai et al., who for the first time demonstrated that ADPN knockout mice exhibited enlarged brain infarct size and worsened neurological deficits following ischemia-reperfusion compared with the wild-type littermates [6]. However, the therapeutic effect of ADPN on ischemic stroke was not examined. Therefore, we initiated ADPN treatment from 6 h after reperfusion and found that multiple doses of ADPN administration could markedly reduce the brain damage after experimental stroke. And the antiapoptotic effect of ADPN when treated after cerebral ischemia corroborated its therapeutic role. The long-term outcome of mice treated with ADPN after reperfusion in further strengthens its effectiveness in stroke treatment. Thus, we for the first time confirmed the therapeutic effect of ADPN after stroke, and the time window could be as long as 6 h after reperfusion.

As the injury secondary to reperfusion is not completely consistent with the primary ischemia injury, the molecular mechanism for ADPN treatment after stroke could be different from its pretreatment before ischemia. Therefore, we focused on the oxidative stress which plays an important role in brain damage [27] including stroke [28] since antioxidant effect is the very common property of ADPN. It has been demonstrated that the oxidative products MDA, 8-OHdG, the carbonyl content, and the nitrotyrosine protein content ould be used for indicators of oxidative stress level and were increased in the pathogenesis of cerebral ischemia-reperfusion injury [29, 30]. We then analyzed these products' content after treatment with ADPN and found that ADPN effectively mitigated oxidative stress as evidenced by MDA, 8-OHdG, the carbonyl content, and the nitrotyrosine protein content. Given that oxidative stress is an important cause of cell deaths and the antiapoptotic effect of ADPN treatment after experimental stroke, the mitigated oxidative stress by ADPN provides further strengths for its therapeutic potential.

Although the antioxidant effect of ADPN was well studied, the exact molecular mechanism of ADPN was unknown, especially in its neuroprotective effect on stroke. HIF-1*α* was an important regulatory node in reducing oxidative stress and inflammation in stroke [17] and was activated after cerebral ischemia [31]. As the key regulator of the transcriptional response to low-oxygen conditions in mammalian cells under both physiological and pathophysiological circumstances [32], HIF-1*α* is essential for the activation of endogenous neuroprotective mechanisms [33]. In fact, HIF-1*α* has been proposed as a potential target for neurodegenerative diseases [32], since it regulates the expression of a broad range of genes that facilitate cellular adaptation to low-oxygen conditions [32]. Furthermore, it has been shown that neuron-specific knockdown of HIF-1*α* increases tissue damage and reduces the survival rate of mice subjected to MCAO [17]. Indeed, HIF-1*α* has been reported to protect neurons from apoptosis caused by oxidative stress [34]. As we recently found out that HIF-1*α* was rapidly stimulated in the penumbra after ischemic stroke [18], we thought that HIF-1*α* might be involved in the treatment effect of ADPN after ischemic stroke. The results of our study revealed that ADPN treatment increased HIF-1*α* expression in neurons following ischemia stroke, and by using AAV-delivered HIF-1*α* siRNA to neurons, we proved that HIF-1*α* was involved in ADPN-induced antioxidant and antiapoptotic effect. Nevertheless, several studies reported opposite effects of HIF-1*α* in cerebral ischemia. For instance, it was reported that HIF-1*α* increased apoptosis that led to ischemia-induced delayed neuronal death and HIF-1*α* deficiency reduced ischemia damage [35, 36]. The discrepancy in findings between these studies may reflect their use of different timings of interventions and multiple roles of HIF-1*α* in brain damage process after stroke.

Another question is how HIF-1*α* leads to the regulation of antioxidant function. This could be explained by its target gene products EPO and VEGF [37]. In the present study, we detected the levels of EPO and VEGF, and our results revealed that ADPN increased EPO and VEGF at 72 h after MCAO, which means that ADPN induces both the expression and activity of HIF-1*α*. Several studies reported that EPO may protect cells by reducing oxidative stress. EPO could exert its antioxidant effect directly by acting on HO-1 and also indirectly by acting on depleting body iron [38]. And exogenous EPO exerted neuroprotection through antioxidant activity after ischemia. In this study, we proved that ADPN increased EPO, suggesting that the antioxidant effect of ADPN may be related to EPO which was regulated HIF-1*α*. Although previous studies confirmed that HIF-1*α*/VEGF pathway plays an important role in promoting angiogenesis and cerebral functional repairment, recent studies show that VEGF had antioxidant effects [39], suggesting VEGF could be another important mediator of HIF-1*α*-regulated antioxidant response. In this study, the results showed that ADPN increased both EPO and VEGF by HIF-1*α*. However, which of the two was more important was undetermined and whether other reaction processes like anaerobic metabolism or inflammatory response was also involved in the effect of ADPN treatment remains to be further confirmed by experiments.

Many studies indicated that angiogenesis was beneficial for the stroke-injured brain, as high levels of new vessel formation following stroke were correlated with better functional recovery and prolonged survival. Angiogenesis was continued for 4 weeks in the penumbra after ischemia [40]. It was reported that overexpression of ADPN promoted angiogenesis in the mouse brain following MCAO [41], even the same results were found in aged mice [5]. The present study demonstrated that exogenous ADPN promoted angiogenesis and reduced brain atrophy 28 days after MCAO. Although we did not demonstrate the mechanism of exogenous ADPN on angiogenesis during ischemia, it is possibly related to the HIF-1*α*/VEGF signaling, as it was reported that ADPN could promote angiogenesis via VEGF in human chondrosarcoma [42].

However, there are always limitations in studies. Since the knockout mice were not used in the present study, this might somewhat cause biased conclusions. To obtain higher validity, further experiments should include conditional gene knockout mice. Also, the effect of ADPN should be testified in both aged and female mice or even mice with hypertension, for its therapeutic potential. Other limitations include lack of confirming the antioxidant function of EPO and VEGF and lack of examination for receptors of ADPN. Also, the exact molecular mechanism by which ADPN stimulated HIF-1*α* in ischemia penumbra was unknown, whether the ADPN receptor or other signaling pathway took part in this needs further exploration.

In conclusion, the present study demonstrated that ADPN treatment is effective in attenuation of cerebral ischemia-reperfusion injury, and HIF-1*α*-regulated antioxidant response is the main molecular mechanism responsible for ADPN treatment for stroke.

## Data Availability

The data used to support the findings of this study are included within the article, and further details are available from the corresponding author upon request.

## Supplementary Materials

Supplementary materials Figure S1: the effect of control AAV virus or HIF-1*α* siRNA AAV virus. (A) Representative photographs of AAV virus expression and (B) the effect verification of HIF-1*α* siRNA by WB. Data are presented as the mean ± SD and analyzed by *t*-test followed by Mann–Whitney test. ^∗^*P* < 0.05 compared to ADPN group, *n* = 4 per group.
