# Supplementary material for: Adiponectin Treatment Attenuates Cerebral Ischemia-Reperfusion Injury through HIF-1α-Mediated Antioxidation in Mice
Source: Oxid Med Cell Longev. 2021 Jul 14;2021:5531048. doi: 10.1155/2021/5531048 (PMC8298180; doi:10.1155/2021/5531048)
Supplement: Supplementary materials — Figure S1: the effect of control AAV virus or HIF-1α siRNA AAV virus. (A) Representative photographs of AAV virus expression and (B) the effect verification of HIF-1α siRNA by WB. Data are presented as the mean ± SD and analyzed by t-test followed by Mann–Whitney test. ∗P < 0.05 compared to ADPN group, n = 4 per group. [file 5531048.f1.docx]

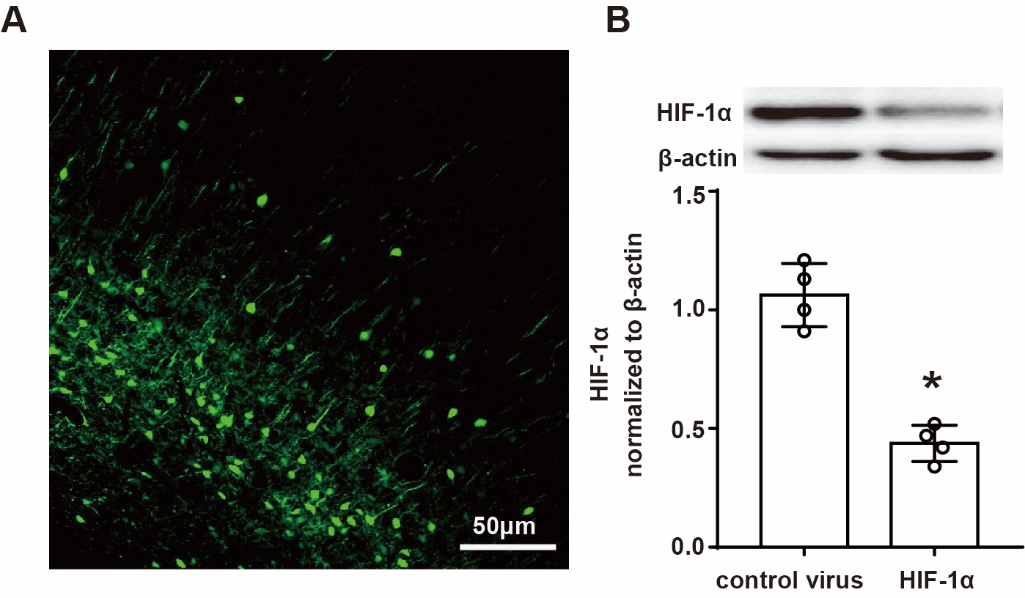


Fig S the effect of control AAV virus or HIF-1α siRNA AAV virus. A representative photographs of AAV virus expression, B the effect verification of HIF-1α siRNA by WB. Data are presented as the mean ± SD and analyzed by t-test followed by Mann Whitney test. **P* < 0.05 compared to ADPN group, *n* = 4 per group.
